# Multivariate Analysis and Health Risk Assessment of Hazardous Trace Elements in Honey from Ordu, Türkiye

**DOI:** 10.1007/s12011-026-05179-2

**Published:** 2026-06-13

**Authors:** Serkan Guney, Saliha Guney, Seydahmet Cay

**Affiliations:** 1 Chemistry and Chemical Processing Technologies, Ulubey Vocational School, Ordu University, Ulubey, Ordu 52000 Turkey; 2 Department of Environmental Engineering, Faculty of Engineering, Giresun University, Gure, Giresun, 28200 Turkey

**Keywords:** Honey, Physicochemical properties, Trace elements, ICP-MS, Principal component analysis, Health risk assessment, Food safety, Bioindicator

## Abstract

Honey is increasingly recognized as both a high-value food product and a sensitive bioindicator of environmental contamination. Here, we provide an integrated assessment of physicochemical properties, trace element composition, and human health risks in honey collected from eight districts of Ordu Province, Türkiye. Significant regional variability (*p* < 0.05) was observed, driven by geographical origin, environmental conditions, and botanical diversity. Multivariate analysis explained 83.6% of total variance and clearly separated samples according to geogenic (Fe–Ni–Cu) and anthropogenic (Cd–Pb–Zn) signatures. Despite detectable spatial variation in trace element concentrations, toxic elements such as As, Cd, and Pb remained at low levels, and carcinogenic risk (CR) values were within acceptable limits. However, chromium emerged as the dominant contributor to non-carcinogenic risk indices (THQ and HI), highlighting its critical role in exposure assessment. Importantly, this contribution is likely conservative, as risk calculations were based on total chromium without speciation, potentially overestimating the actual toxicological impact relative to hexavalent chromium [Cr(VI)]. These findings demonstrate that honey from the studied region is safe for consumption while revealing that chromium-driven risk signals may mask the true exposure profile if speciation is not considered. More broadly, this study establishes a robust framework integrating chemometric analysis and risk assessment, positioning honey as a powerful tool for environmental monitoring and food safety evaluation.

**Graphical Abstract:**

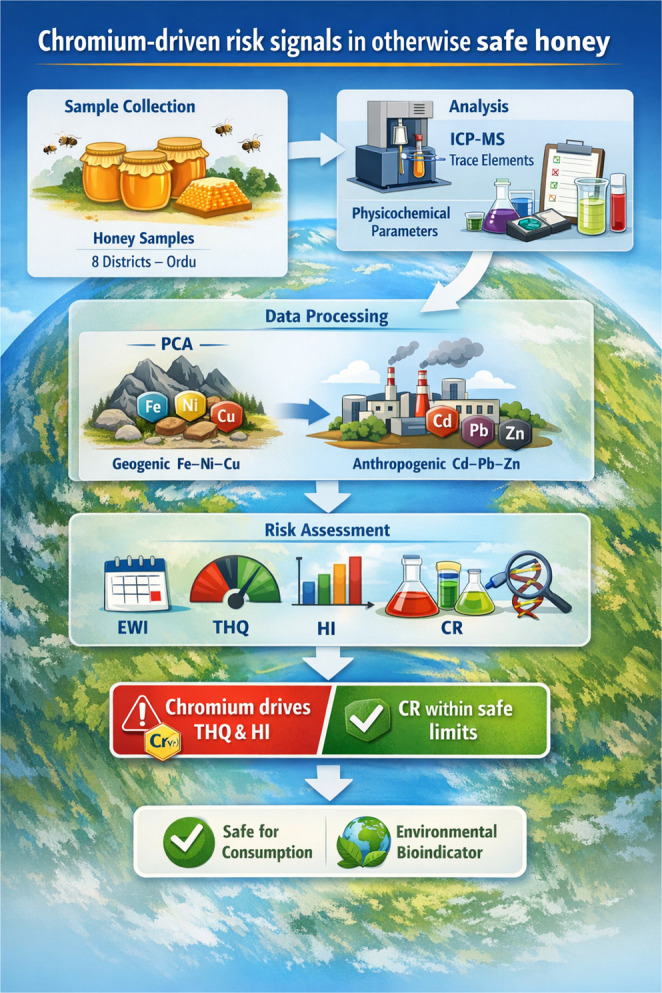

## Introduction

Beekeeping is an important agricultural activity that produces high-value products such as honey, pollen, royal jelly, propolis, and bee venom, as well as biological materials including queen bees, swarms, and package bees [1]. It does not require direct land use and can be integrated with various agricultural systems, making it a flexible and sustainable production practice. Furthermore, interactions between beekeeping and plant production systems enhance agricultural sustainability by improving pollination efficiency and supporting biodiversity [2].

Turkey is highly suitable for apiculture due to its diverse climatic conditions, rich flora, and genetically diverse honey bee (*Apis mellifera*) populations [3]. Beekeeping in Turkey plays a significant role in maintaining ecological balance and supporting agricultural productivity through effective pollination services [2, 4]. According to FAO data, Turkey is among the leading honey-producing countries worldwide.

According to FAO statistics, Turkey ranked second in the world in 2022 with a total honey production of 118,297 tons, following China. Based on FAO’s 2022 global data, the average honey yield per hive is approximately 18.1 kg. Although India is the world leader in terms of the number of beehives, its yield per hive remains only 5.9 kg. In contrast, China—the second country in terms of hive numbers—has a significantly higher yield of 46.9 kg per hive. Turkey ranks third globally in terms of hive count, with an average honey yield of 13.2 kg per hive.

Honey is a natural sweet substance whose composition is strongly influenced by botanical origin and environmental conditions. In addition to its nutritional value, honey contains various bioactive and mineral components that contribute to its functional properties [5, 6]. However, its composition may also be affected by environmental contamination. Trace elements present in honey originate from both natural sources and anthropogenic activities, including industrial emissions, agricultural practices, and traffic-related pollution.

Honey contains essential elements such as Na, Fe, Cu, Zn, and Ni, which play important roles in physiological processes, including enzyme activity, oxygen transport, and metabolic regulation [5]. However, excessive exposure to these elements may lead to toxic effects. In addition, honey may contain potentially toxic elements such as Al, As, Cd, Pb, Hg, and Tl, which have no known biological function and may pose serious health risks even at low concentrations [7, 8].

Due to their wide foraging range, bees can accumulate environmental contaminants from nectar, pollen, and water sources and transfer them into honey. Therefore, honey can serve as a bioindicator of environmental pollution and a potential source of human exposure to trace elements [9–11].

Unlike previous studies focusing solely on either physicochemical characterization or trace element contamination, this study integrates comprehensive compositional analysis with multivariate statistical modeling and human health risk assessment to provide a holistic evaluation of honey quality. In addition, the study highlights the potential overestimation of chromium-related health risks due to the use of total chromium concentrations, emphasizing the importance of metal speciation in future risk assessment studies. This combined approach offers a more reliable framework for assessing both food quality and environmental exposure using honey as a bioindicator.

In addition to regional characterization, this study provides a comparative perspective by evaluating the obtained trace element concentrations in relation to previously reported data from different geographical regions and time periods. This approach enhances the interpretative power of the results and contributes to a broader understanding of spatial and temporal trends in honey contamination.

## Materials and Methods

### Honey Samples

**Fig. 1:**
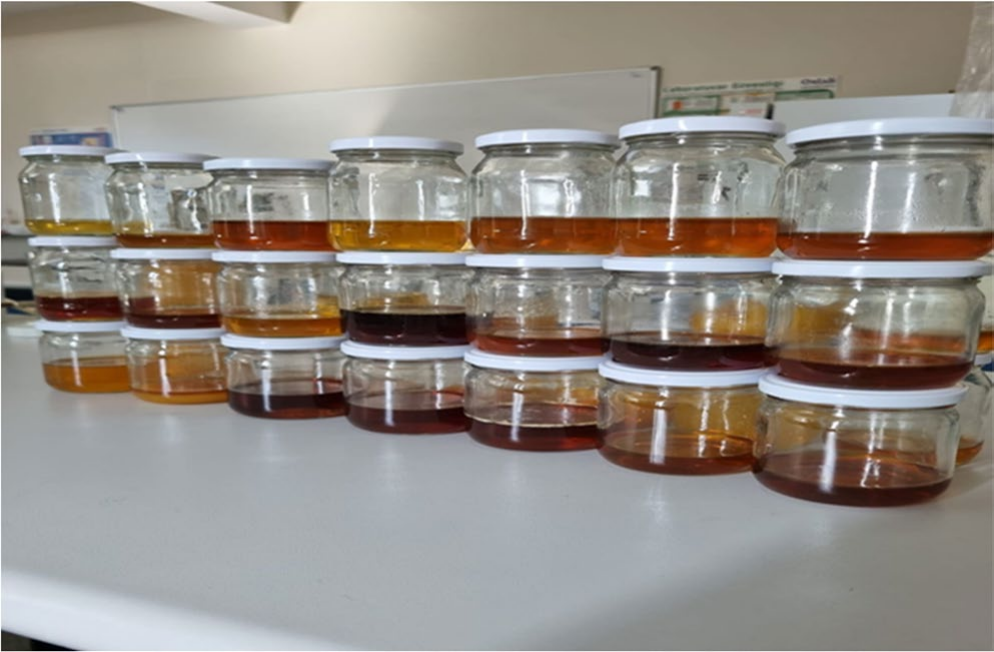
Honey samples

A total of 24 honey samples were collected from eight different districts of Ordu province, Turkey: Gürgentepe, Ulubey, Çatalpınar, Gölköy, Kabataş, Perşembe, Ünye, and Gülyalı. To ensure representative sampling, three separate beekeeping establishments registered in the official Beekeeping Registration System (BRS) database were selected from each district. As shown in Fig. 1, the honey samples were carefully packaged in clean glass jars and stored at 4 °C until ready for analysis.

**Fig. 2:**
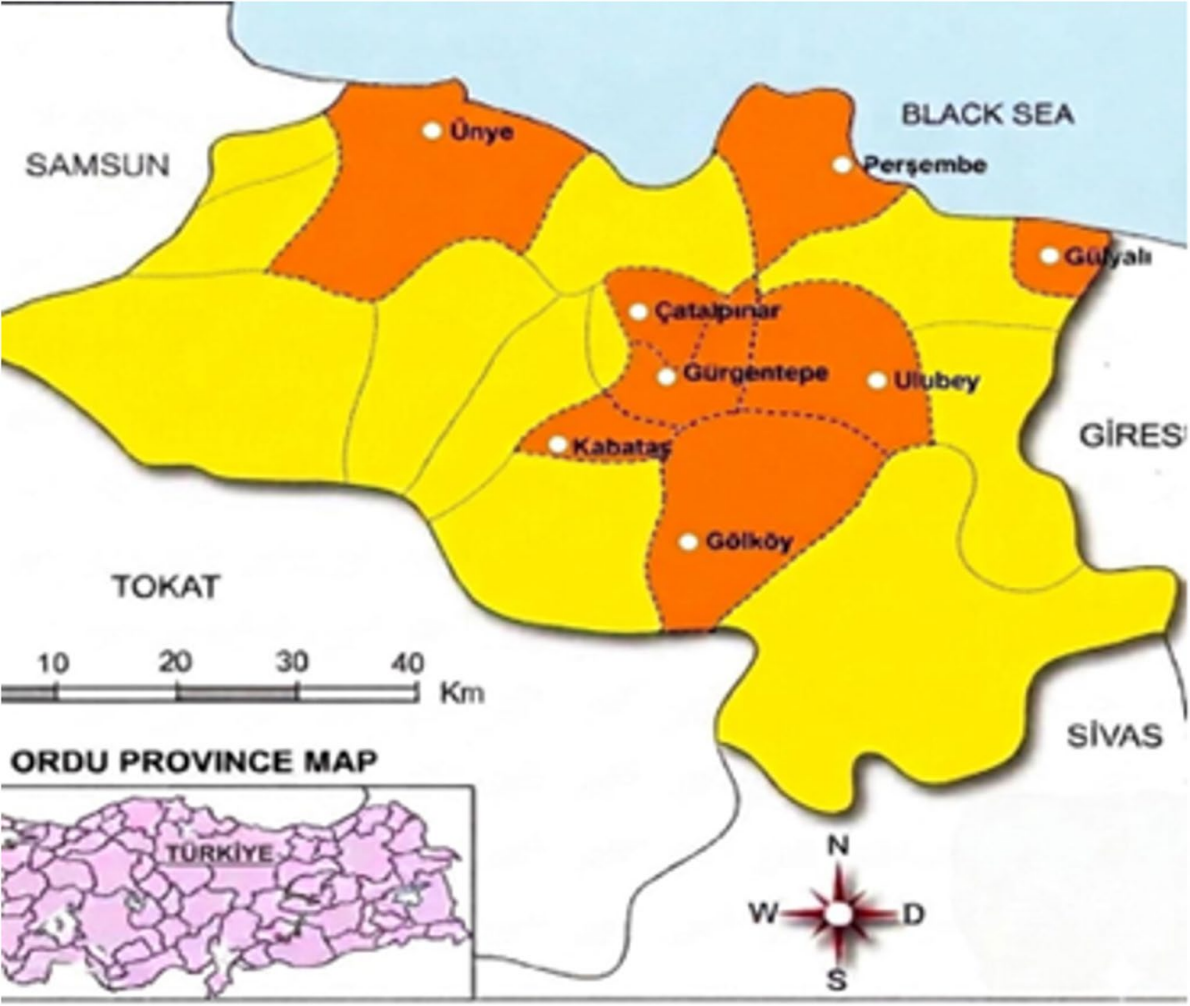
Locations of honey samples

Ordu province has a surface area of 5952 km², and its districts vary in altitude from sea level to 1250 m. The altitudes of the districts from which honey samples were taken are: Gürgentepe 1235 m, Ulubey 550 m, Çatalpınar 150 m, Gölköy 850 m, Kabataş 430 m, Perşembe 100 m, Ünye 20 m, and Gülyalı 100 m. The locations where honey samples were collected are presented on the map in Fig. 2.

### Determination of the Physical Properties of Honey Samples

#### Determination of Moisture Content of Honey Samples

After the honey samples were thoroughly homogenized, their moisture content was measured using an Abbe refractometer according to the TS 13,365 standard. The obtained data were evaluated based on the correlation between the refractive index and the water content of the honey, and the results were reported in g/100g [12].

#### Determination of pH and Free Acidity

The free acidity (FA) of the honey samples was determined by titrimetric analysis according to the TS 13,360 standard. A 10 g honey sample was dissolved in 75 mL of distilled water and titrated with 0.1 N NaOH solution until the pH reached 8.3. The free acidity, expressed as milliequivalents of acid per kilogram of honey (meq/kg), was calculated using the following formula:

$$FA=\left(V2-V1\right).N.1000/m$$

Where:

FA: Free acidity (meq/kg).

V2: Volume of NaOH used for the sample (mL).

V1: Volume of NaOH used for the blank (mL).

N: Normality of the NaOH solution (0.1 N).

m: Mass of the honey sample (g).

#### Ash Determination in Honey Samples

Ash determination was performed by placing 2 g of honey sample into tared crucibles and subjecting them to heat treatment in an ash furnace at 550 °C until white ash was formed. Afterwards, the crucibles were removed from the ash furnace, placed in a desiccator, and allowed to cool. Once at room temperature, the crucibles were weighed, and the amount of ash formed was determined. The % ash values were then calculated using the following formula:

$$\begin{array}{c}\%\;Ash\;=(Ash\;formed\;as\;a\;result\;a\;combustion/\\/Amount\;of\;honey\;weighed)\times100\end{array}$$

#### Measurement of Electrical Conductivity in Honey Samples

For electrical conductivity measurements, 20 g of honey were dissolved in a specified amount of pure water, and the resulting solution was transferred to a 100 mL volumetric flask, then diluted with pure water. 40 mL of this solution was taken and transferred to an Erlenmeyer flask, and brought to a reference temperature of 20 °C using a water bath. The remaining solution was used to clean the conductivity electrode. The electrode was connected to the conductivity measuring device, immersed in the solution, and the conductivity value of the solution was measured [13].

#### Color Determination in Honey Samples

Color measurements were performed using a color photometer (Hanna, HI 96785, USA) that compared the light transmittance of honey to analytical-grade glycerol. Approximately 2 g honey samples were heated in an ultrasonic bath at 35 °C for several minutes. The liquid honey, free of air bubbles, was transferred to a plastic cuvette and the color was read. Color grades were expressed in millimeters (mm) on the Pfund scale [14].

#### Hazardous Trace Element Analysis

The determination of hazardous trace elements was performed using an inductively coupled plasma mass spectrometer (ICP-MS, Thermo Scientific iCAP-Q). Prior to analysis, honey samples were subjected to microwave-assisted acid digestion to ensure complete mineralization.

Approximately 0.5 g of homogenized honey sample was accurately weighed into Teflon digestion vessels. A mixture of 9 mL of concentrated nitric acid (HNO₃, 65%, suprapure grade) and 1 mL of hydrogen peroxide (H₂O₂, 30%) was added. The digestion process was carried out using a microwave digestion system (Milestone Ethos Easy) under controlled conditions (ramp to 200 °C within 15 min and hold for 20 min).

After digestion, the samples were cooled to room temperature and quantitatively transferred into 50 mL polypropylene tubes and diluted to volume with ultrapure water (resistivity 18.2 MΩ·cm). Blank samples were prepared using the same procedure.

Calibration curves were established using multi-element standard solutions (Merck ICP multi-element standard) at appropriate concentration ranges. The linearity of calibration curves (R² > 0.999) was verified for all analyzed elements.

Quality assurance and quality control (QA/QC) procedures included:

- Analysis of procedural blanks.
- Triplicate measurements of each sample.
- Use of certified reference material (CRM, UME CRM 1201).
- Determination of limits of detection (LOD) and quantification (LOQ), calculated as 3σ and 10σ of blank signals, respectively.

Instrument operating conditions (RF power, nebulizer gas flow, dwell time) were optimized daily to ensure analytical accuracy and precision. To ensure analytical reliability, ICP-MS measurements included isotope-specific monitoring, internal standard correction, helium collision cell operation, and certified reference material validation. Measured CRM concentrations showed good agreement with certified values, with recoveries ranging between 92% and 105%, confirming acceptable analytical accuracy for trace-level determination. All measurements were expressed as mg/kg (wet weight basis).

### Estimation of Health Risks

The potential health risks associated with metal exposure through honey consumption were evaluated using Estimated Daily Intake (EDI), Target Hazard Quotient (THQ), Hazard Index (HI), and Cancer Risk (CR) models. Two consumption scenarios were considered in this study: once-weekly and three-times-weekly honey consumption.

The EDI value was calculated according to the formula suggested by USEPA and other authors [15].

1 $$EDI=\left(E_c\times D_c\right)/B_W$$

where E_c_ refers to the element concentration (mg/kg) in honey, D_c_ is the daily honey consumption (kg/day), and B_w_ is the weight (70 kg for adults). For the evaluated scenarios, weekly honey consumption amounts were normalized to daily intake equivalents. The calculated EDI values were used for subsequent toxicological risk calculations.

The non-carcinogenic risk associated with metal exposure was evaluated using the Target Hazard Quotient (THQ) according to the methodology recommended by the United States Environmental Protection Agency:

2 *THQ=EDI/RfD*

where RfD represents the oral reference dose for each metal (mg kg⁻¹ day⁻¹). A THQ value lower than 1 indicates that adverse non-carcinogenic health effects are unlikely, whereas THQ values greater than 1 may indicate potential health concerns [16].

The overall non-carcinogenic health risk associated with simultaneous exposure to multiple metals was evaluated using the Hazard Index (HI), defined as the sum of individual THQ values:

3 $$\:\mathrm{HI=}\sum\:_{i=1}^{n}\mathrm{T}\mathrm{H}{\mathrm{Q}}_{i}$$

where THQ_i_ represents the THQ value of each individual metal. HI values below 1 indicate that the cumulative non-carcinogenic risk is considered negligible.

The carcinogenic risk associated with trace-element exposure through honey consumption was estimated using the Cancer Risk (CR) model:

4 *CR=EDI× CsF*

where CsF is the cancer slope factor ((mg kg⁻¹ day⁻¹)⁻¹). The CsF values used in this study were obtained from the United States Environmental Protection Agency (Cr: 0.5; Ni: 1.7; Pb: 0.0085; As: 1.5). A CR value between 10⁻⁶ and 10⁻⁴ is generally considered an acceptable carcinogenic risk range according to USEPA guidelines [17].

### Data Analysis

Statistical analyses were carried out to evaluate the variation among the sample variables. All statistical calculations were performed using the Statistical Package for the Social Sciences (SPSS, version 26.0; IBM Corp., Armonk, NY, USA). Prior to inferential statistical analyses, data normality and homogeneity of variances were evaluated using the Shapiro–Wilk and Levene’s tests, respectively. For variables meeting parametric assumptions, one-way analysis of variance (ANOVA) followed by Tukey’s HSD post-hoc test was performed to determine significant differences among districts. For variables that did not satisfy parametric assumptions, the Kruskal–Wallis test was applied. Statistical significance was accepted at *p* < 0.05. The results were expressed as mean ± standard deviation (SD) along with the minimum and maximum values to describe the distribution of the data. In addition, principal component analysis (PCA) was applied to identify possible relationships and patterns among the variables. For data visualization and multivariate analysis, several R packages, including *factoextra*, *rioja*, *pheatmap*, *tidyverse*, *ggplotify*, *heatmaply*, *gplots*, and *plotly*, were utilized to generate PCA plots and heatmap diagrams. These approaches allowed a clearer interpretation of the relationships between honey samples and metal variables.

## Results

### Physical Properties of Honey Samples

The physicochemical properties of honey samples collected from different districts of Ordu province, including moisture content, pH, free acidity, ash content, electrical conductivity, and color, were comprehensively evaluated, and the results are presented in Table 1. Significant variations were observed among districts, highlighting the strong influence of geographical origin and botanical diversity on honey composition. These differences not only reflect variations in quality and maturity but also indicate distinct mineral and compositional profiles, providing a reliable basis for the differentiation of honey samples according to their regional characteristics.

**Table 1:** Concentrations of hazardous trace elements in honey samples from different districts of Ordu province (ppm)

Location	Moisture (%)	Brix (%)	pH	Free Acidity	Ash (%)	EC	Color
Perşembe	16.33 ± 1.79ᵃ	81.92 ± 1.66ᵃ	4.67 ± 0.88ᵃᵇ	21.79 ± 12.56ᵃ	0.31 ± 0.22ᵃ	574.67 ± 604.31ᵃᵇ	69.00 ± 37.72ᵃ
Ulubey	18.07 ± 1.89ᵃ	80.33 ± 1.84ᵃ	4.25 ± 0.51ᵃ	19.25 ± 1.81ᵃ	0.35 ± 0.18ᵃ	547.00 ± 498.83ᵃ	60.00 ± 31.00ᵃ
Gölköy	16.93 ± 1.47ᵃ	81.33 ± 1.42ᵃ	3.78 ± 0.08ᵃ	21.11 ± 4.75ᵃ	0.10 ± 0.05ᵃ	271.00 ± 37.24ᵃ	29.67 ± 7.23ᵃ
Gülyalı	15.13 ± 0.64ᵃ	83.08 ± 0.80ᵃ	4.22 ± 0.78ᵃ	21.77 ± 6.55ᵃ	0.40 ± 0.28ᵃ	341.60 ± 222.43ᵃ	68.00 ± 4.36ᵃ
Çatalpınar	17.33 ± 1.10ᵃ	81.00 ± 1.09ᵃ	4.18 ± 0.19ᵃ	26.90 ± 3.69ᵇ	0.26 ± 0.16ᵃ	549.33 ± 177.47ᵃ	76.33 ± 5.51ᵃ
Kabataş	18.67 ± 1.15ᵃ	79.58 ± 1.15ᵃ	4.26 ± 0.41ᵃ	16.69 ± 0.91ᵃ	0.20 ± 0.08ᵃ	459.67 ± 279.20ᵃ	77.67 ± 13.50ᵃ
Ünye	16.73 ± 1.47ᵃ	81.50 ± 1.32ᵃ	3.85 ± 0.13ᵃ	15.98 ± 2.80ᵃ	0.06 ± 0.01ᵃ	194.17 ± 20.25ᵃ	42.67 ± 23.97ᵃ
Gürgentepe	18.67 ± 1.89ᵃ	79.58 ± 1.84ᵃ	4.81 ± 0.31ᵇ	17.89 ± 4.97ᵃ	0.36 ± 0.20ᵃ	1043.00 ± 615.58ᵇ	91.33 ± 43.32ᵃ

*Values are expressed as mean ± standard deviation. Different letters in the same column indicate significant differences between locations according to Duncan’s Multiple Range Test (*p* < 0.05)

The comprehensive evaluation of physicochemical properties revealed significant regional variations among honey samples. Moisture content ranged from 15.13% in Gülyalı to 18.67% in Kabataş and Gürgentepe, while Brix values showed an inverse trend, confirming the well-established negative relationship between water content and soluble solids [18]. Gülyalı samples were characterized by low moisture and high Brix values, indicating higher maturity and concentration, whereas Gürgentepe and Kabataş samples exhibited higher moisture and lower Brix values, suggesting relatively diluted honey composition.

The pH and free acidity values were within acceptable limits for all samples, indicating good quality and absence of undesirable fermentation. Çatalpınar samples showed higher acidity, reflecting increased organic acid content, while Ünye samples exhibited lower acidity values. Ash content and electrical conductivity, both indicators of mineral composition, were notably higher in Gürgentepe and Ulubey samples, supporting the presence of mineral-rich floral sources [19]. Electrical conductivity values were strongly associated with ash content, consistent with previous findings [20].

Color analysis further supported these observations, as darker honeys (Gürgentepe and Kabataş) corresponded to higher mineral content and conductivity, whereas lighter honeys (Gölköy and Ünye) exhibited lower values. The observed variability among districts highlights the significant influence of botanical origin, environmental conditions, and geographical diversity on honey composition. Overall, all samples complied with international quality standards, confirming their suitability for consumption while demonstrating distinct regional characteristics.

### Hazardous Trace Element Concentrations in Honey

Due to increasing industrialization and intensified agricultural activities in recent decades, environmental pollution has become a major global concern. Hazardous trace elements present in the environment can be transferred to honey through contaminated soil, water, air, and plant nectar sources. During foraging, bees collect nectar and pollen from plants exposed to these contaminants, which may lead to the accumulation of hazardous trace elements in honey. Because honey is widely consumed worldwide due to its nutritional and medicinal properties, the presence of hazardous trace elements may pose potential risks to human health and food safety. Moreover, honey and bee products are considered useful bioindicators of environmental contamination, reflecting the quality of the surrounding ecosystem [5, 21].

Honey samples collected from eight districts of Ordu province (Ulubey, Gölköy, Gürgentepe, Perşembe, Kabataş, Ünye, Gülyalı and Çatalpınar) were measured for hazardous trace elements, including As, Pb, Cd, Ni, Zn, Se, Cr, Cu, Fe and Mn, using ICP-MS, and the results are presented in Table 2. The comparison of trace metal concentrations among the investigated regions revealed noticeable spatial variability, indicating that environmental and geographical factors may strongly influence the elemental composition of honey. In particular, the honey samples collected from Gülyalı exhibited markedly elevated concentrations of Fe and Ni compared to other locations. Such enrichment may be associated with local geological characteristics or anthropogenic activities, including traffic emissions and agricultural inputs, which have been reported as potential sources of trace metals in apicultural environments. Similarly, the Ulubey samples showed relatively high levels of Zn and Mn, suggesting a possible contribution from soil mineral composition and plant uptake processes, since these elements are commonly absorbed by plants and transferred into nectar during honey production. Previous studies have demonstrated that regional variations in soil composition, vegetation type, and environmental pollution significantly affect the trace metal profile of honey, making honey a useful indicator of local environmental conditions [7, 15].

**Table 2:** Concentrations of hazardous trace elements in honey samples from different districts of Ordu province (ppm)

Location	Cr	Mn	Fe	Ni	Cu	Zn	As	Cd	Pb	Se
Kabataş	51.04 ± 1.73ᵇ	284.93 ± 19.89ᵇ	225.86 ± 11.56ᶠ	2.33 ± 0.62ᶜ	2.65 ± 0.29ᵈ	14.96 ± 4.55ᶜ	0.00 ± 0.00ᵇ	0.70 ± 0.02ᵇ	0.70 ± 0.28ᵇ	< LOD
Gülyalı	54.91 ± 0.44ᵇ	70.64 ± 5.30ᶠ	3759.16 ± 404.56ᵃ	65.47 ± 90.29ᵃ	15.55 ± 22.76ᵃ	5.77 ± 4.32ᵈ	0.03 ± 0.01ᵃ	0.36 ± 0.01ᶜ	0.36 ± 0.15ᶜ	< LOD
Ulubey	72.04 ± 3.05ᵃ	311.58 ± 40.08ᵇ	1839.45 ± 162.71ᵇ	14.20 ± 11.45ᵇ	9.86 ± 7.39ᵇ	52.15 ± 76.99ᵇ	0.01 ± 0.00ᵇ	0.81 ± 0.55ᵇ	0.81 ± 0.55ᵇ	< LOD
Perşembe	46.68 ± 0.88ᶜ	41.98 ± 4.09ᶠ	852.15 ± 100.26ᶜ	6.58 ± 6.79ᶜ	10.15 ± 15.37ᵇ	8.67 ± 3.24ᶜ	0.03 ± 0.01ᵃ	0.29 ± 0.01ᶜ	0.29 ± 0.14ᶜ	< LOD
Çatalpınar	56.24 ± 0.98ᵇ	513.03 ± 10.08ᵃ	1567.03 ± 210.53ᵇ	3.89 ± 2.09ᶜ	2.80 ± 0.43ᵈ	8.99 ± 5.31ᶜ	0.00 ± 0.00ᵇ	0.36 ± 0.01ᶜ	0.36 ± 0.17ᶜ	< LOD
Gölköy	80.03 ± 1.78ᵃ	50.05 ± 2.61ᶠ	401.19 ± 38.42ᵉ	3.40 ± 1.11ᶜ	2.20 ± 0.71ᵈ	13.33 ± 9.79ᶜ	0.00 ± 0.00ᵇ	0.48 ± 0.01ᵇ	0.48 ± 0.09ᵇ	< LOD
Ünye	59.03 ± 0.38ᵇ	19.76 ± 0.37ᵍ	9.03 ± 1.56ᵍ	3.37 ± 0.76ᶜ	1.67 ± 0.31ᵉ	2.88 ± 0.68ᵉ	0.00 ± 0.00ᵇ	0.39 ± 0.01ᶜ	0.39 ± 0.11ᶜ	< LOD
Gürgentepe	62.39 ± 1.33ᵇ	435.83 ± 29.36ᵃ	280.09 ± 48.51ᶠ	4.23 ± 1.31ᶜ	3.18 ± 1.33ᵈ	114.12 ± 90.69ᵃ	0.10 ± 0.07ᵃ	1.79 ± 2.16ᵃ	1.79 ± 2.16ᵃ	< LOD

*Values are expressed as mean ± standard deviation. Different letters in the same column indicate significant differences between locations according to Duncan’s Multiple Range Test (*p* < 0.05)

A particularly noteworthy observation was the comparatively high Pb and Zn concentrations detected in the Gürgentepe samples, which may indicate localized environmental contamination. Lead contamination in honey has frequently been linked to atmospheric deposition from traffic emissions, industrial activities, and urbanization processes. Bees can collect contaminants from a foraging radius of several kilometers, allowing honey to reflect the cumulative environmental exposure of the surrounding ecosystem. Although the concentrations of Cd and As remained relatively low in most samples, the presence of Pb in certain regions highlights the importance of continuous monitoring due to its potential health implications. Chronic exposure to heavy metals such as Pb and Cd has been associated with neurological, renal, and cardiovascular disorders, particularly in vulnerable populations such as children and pregnant women. Nevertheless, the detected concentrations generally fall within ranges reported in recent international studies, suggesting that the analyzed honey samples are unlikely to pose significant health risks under normal consumption patterns [22, 23].

### Assessing Health Risks

To evaluate the potential human health risks associated with hazardous trace element concentrations in honey collected from different districts of Ordu province, several widely used risk assessment indices were calculated. These included the EDI (Table 3), THQ (Table 4), HI (Table 5), and CR (Table 6). These parameters were estimated based on the assumption that adults consume honey once or more than once per week. The applied methodology follows commonly used human health risk assessment approaches recommended by the United States Environmental Protection Agency and previously applied in studies evaluating trace element exposure through food consumption [24, 25].

**Table 3:** Estimated Daily Intake (EDI, mg kg⁻¹ bw day⁻¹) derived from weekly honey consumption scenarios

Location	Cr	Mn	Fe	Ni	Cu	Zn	As	Cd	Pb	Se
Kabataş	0.0030/0.0089	0.0127/0.0382	0.0751/0.2252	0.00058/0.00174	0.00040/0.00121	0.00213/0.00639	< LOD	0.00003/0.00010	0.00003/0.00010	< LOD
Gülyalı	0.00190/0.00571	0.00171/0.00514	0.0348/0.1043	0.00027/0.00081	0.00041/0.00124	0.00035/0.00106	< LOD	0.00001/0.00004	0.00001/0.00004	< LOD
Ulubey	0.00230/0.00689	0.0209/0.0628	0.0640/0.1919	0.00016/0.00048	0.00011/0.00034	0.00037/0.00110	< LOD	0.00001/0.00004	0.00001/0.00004	< LOD
Perşembe	0.00327/0.00980	0.00204/0.00613	0.0164/0.0491	0.00014/0.00042	0.00009/0.00027	0.00054/0.00163	< LOD	0.00002/0.00006	0.00002/0.00006	< LOD
Çatalpınar	0.00241/0.00723	0.00081/0.00241	0.00037/0.00111	0.00014/0.00041	0.00007/0.00020	0.00012/0.00035	< LOD	0.00002/0.00005	0.00002/0.00005	< LOD
Gölköy	0.00254/0.00764	0.0178/0.0534	0.0114/0.0343	0.00017/0.00052	0.00013/0.00039	0.00466/0.01397	0.00000/0.00001	0.00007/0.00022	0.00007/0.00022	< LOD
Ünye	0.00294/0.00881	0.0127/0.0382	0.0751/0.2252	0.00058/0.00174	0.00040/0.00121	0.00213/0.00639	< LOD	0.00003/0.00010	0.00003/0.00010	< LOD
Gürgentepe	0.00190/0.00571	0.00171/0.00514	0.0348/0.1043	0.00027/0.00081	0.00041/0.00124	0.00035/0.00106	< LOD	0.00001/0.00004	0.00001/0.00004	< LOD

* Values are expressed as estimated daily intake (EDI, mg kg⁻¹ bw day⁻¹) derived from two weekly honey consumption scenarios: 20 g honey consumed once weekly (1×) and 60 g honey consumed three times weekly (3×), assuming an average adult body weight of 70 kg

**Table 4:** Target Hazard Quotient (THQ) values derived from estimated daily intake (EDI) under different weekly honey consumption scenarios * Values are expressed as THQ derived from EDI calculated from two weekly honey consumption scenarios: 1× = one honey consumption per week (20 g week⁻¹) and 3× = three honey consumptions per week (60 g week⁻¹), assuming an average adult body weight of 70 kg

Location	Cr	Mn	Fe	Ni	Cu	Zn	As	Cd	Pb	Se
Kabataş	0.0992/0.2976	0.0119/0.0356	0.0019/0.0056	0.0001/0.0004	0.0001/0.0002	0.0001/0.0003	0.0000/0.0000	0.0041/0.0122	0.0012/0.0035	< LOD
Gülyalı	0.1067/0.3202	0.0029/0.0088	0.0313/0.0939	0.0019/0.0057	0.0004/0.0012	0.0000/0.0001	0.0006/0.0017	0.0021/0.0063	0.0006/0.0018	< LOD
Ulubey	0.1400/0.4201	0.0130/0.0389	0.0153/0.0460	0.0004/0.0012	0.0003/0.0008	0.0004/0.0011	0.0002/0.0006	0.0047/0.0142	0.0014/0.0040	< LOD
Perşembe	0.0907/0.2722	0.0017/0.0052	0.0071/0.0213	0.0002/0.0006	0.0003/0.0008	0.0001/0.0002	0.0006/0.0017	0.0017/0.0051	0.0005/0.0014	< LOD
Çatalpınar	0.1093/0.3279	0.0214/0.0641	0.0131/0.0392	0.0001/0.0003	0.0001/0.0002	0.0001/0.0002	0.0000/0.0000	0.0021/0.0063	0.0006/0.0018	< LOD
Gölköy	0.1555/0.4666	0.0021/0.0063	0.0033/0.0100	0.0001/0.0003	0.0001/0.0002	0.0001/0.0002	0.0000/0.0000	0.0028/0.0084	0.0008/0.0024	< LOD
Ünye	0.1147/0.3442	0.0008/0.0025	0.0001/0.0002	0.0001/0.0003	0.0000/0.0001	0.0000/0.0001	0.0000/0.0000	0.0023/0.0068	0.0007/0.0019	< LOD
Gürgentepe	0.1213/0.3638	0.0182/0.0545	0.0023/0.0070	0.0001/0.0004	0.0001/0.0002	0.0008/0.0023	0.0019/0.0058	0.0104/0.0313	0.0030/0.0089	< LOD

**Table 5:** Hazard Index (HI) values derived from estimated daily intake (EDI) under different weekly honey consumption scenarios

Location	HI (1×/week)	HI (3×/week)
Kabataş	0.1187	0.3560
Gülyalı	0.1466	0.4398
Ulubey	0.1756	0.5268
Perşembe	0.1028	0.3085
Çatalpınar	0.1467	0.4400
Gölköy	0.1647	0.4941
Ünye	0.1187	0.3559
Gürgentepe	0.1581	0.4743

* Values are expressed as HI derived from estimated daily intake (EDI) calculated from two weekly honey consumption scenarios: 1× = one honey consumption per week (20 g week⁻¹) and 3× = three honey consumptions per week (60 g week⁻¹), assuming an average adult body weight of 70 kg

**Table 6:** Cancer Risk (CR) values derived from estimated daily intake (EDI) under different weekly honey consumption scenarios

Location	As (1×/week)	As (3×/week)	Cr (1×/week)	Cr (3×/week)	Ni (1×/week)	Ni (3×/week)	Pb (1×/week)	Pb (3×/week)	Cd (1×/week)	Cd (3×/week)
Kabataş	0.00E + 00	0.00E + 00	1.49E-06	4.47E-06	6.41E-08	1.93E-07	2.84E-09	8.54E-09	4.43E-07	1.33E-06
Gülyalı	8.74E-07	2.63E-06	1.60E-06	4.79E-06	1.80E-06	5.41E-06	1.46E-09	4.37E-09	2.29E-07	6.86E-07
Ulubey	2.91E-07	8.74E-07	2.10E-06	6.29E-06	3.90E-07	1.17E-06	3.29E-09	9.87E-09	5.01E-07	1.50E-06
Perşembe	8.74E-07	2.63E-06	1.36E-06	4.09E-06	1.83E-07	5.49E-07	1.17E-09	3.51E-09	1.80E-07	5.41E-07
Çatalpınar	0.00E + 00	0.00E + 00	1.64E-06	4.93E-06	1.08E-07	3.24E-07	1.46E-09	4.37E-09	2.29E-07	6.86E-07
Gölköy	0.00E + 00	0.00E + 00	2.33E-06	7.00E-06	9.44E-08	2.83E-07	1.94E-09	5.81E-09	3.04E-07	9.13E-07
Ünye	0.00E + 00	0.00E + 00	1.71E-06	5.16E-06	9.36E-08	2.81E-07	1.59E-09	4.74E-09	2.49E-07	7.46E-07
Gürgentepe	2.91E-06	8.74E-06	1.81E-06	5.43E-06	1.17E-07	3.51E-07	7.11E-09	2.13E-08	1.11E-06	3.33E-06

* Values are expressed as CR derived from estimated daily intake (EDI) calculated from two weekly honey consumption scenarios: 1× = one honey consumption per week (20 g week⁻¹) and 3× = three honey consumptions per week (60 g week⁻¹), assuming an average adult body weight of 70 kg

#### Estimated Daily Intake

The estimated daily intake (EDI) values were calculated to assess the potential dietary exposure to hazardous trace elements through honey consumption. EDI is widely used in food safety and human health risk assessment studies to estimate the amount of a contaminant ingested daily relative to body weight and to evaluate potential risks associated with food consumption according to World Health Organization, Food and Agriculture Organization, and United States Environmental Protection Agency methodologies.

In the present study, EDI values were estimated based on two weekly honey consumption scenarios: one honey consumption per week (20 g week⁻¹) and three honey consumptions per week (60 g week⁻¹) for adults with an average body weight of 70 kg. Weekly consumption amounts were converted to daily intake equivalents prior to toxicological risk calculations.

The calculated EDI values varied depending on the sampling locations and the concentrations of hazardous trace elements in the analyzed honey samples and are presented Table 3. Among the investigated elements, Fe and Mn exhibited relatively higher EDI values, mainly due to their elevated concentrations in some honey samples. However, these elements are considered essential trace elements required for various physiological and metabolic functions in the human body, and the estimated intake levels remained within acceptable ranges. In contrast, toxic elements such as As, Cd, and Pb exhibited very low EDI values, indicating minimal dietary exposure through honey consumption.

Comparison of the obtained EDI values with previously reported studies suggests that honey contributes only a minor proportion of the daily dietary exposure to hazardous trace elements. Similar findings have been reported in previous studies investigating trace-element contamination in honey and other bee products, where estimated intake levels generally remained below toxicological concern thresholds and were therefore not associated with significant health risks [5, 11].

These results indicate that the consumption of honey from the studied districts contributes only a minor portion of the overall dietary exposure to hazardous trace elements, suggesting that honey consumption at the assumed intake levels does not pose a significant health concern.

#### Target Hazard Quotient

The Target Hazard Quotient (THQ) values were calculated to evaluate the potential non-carcinogenic health risks associated with exposure to hazardous trace elements through honey consumption. THQ is a widely used risk assessment index in food safety and toxicological studies and is recommended within the human health risk assessment framework developed by the United States Environmental Protection Agency. According to this approach, THQ values lower than 1 indicate that adverse non-carcinogenic health effects are unlikely, whereas THQ values greater than 1 may indicate potential health concerns.

In the present study, all calculated THQ values for the investigated trace elements remained below the threshold value of 1 under both evaluated consumption scenarios, indicating that honey consumption from the studied districts is unlikely to pose significant non-carcinogenic health risks for adult consumers (Table 4). In particular, the THQ values calculated for potentially toxic elements such as As, Cd, and Pb were extremely low, suggesting minimal exposure through honey consumption. Similar observations have been reported in previous studies investigating trace-element contamination in honey and other bee products [22].

Among the analyzed elements, chromium (Cr) exhibited relatively higher THQ contributions compared with the other investigated elements, particularly under the higher consumption scenario (three honey consumptions per week). However, it is important to emphasize that the chromium-related calculations were based on total chromium concentrations determined by ICP-MS analysis, whereas the toxicological reference dose (RfD) used in risk assessment frameworks is primarily associated with hexavalent chromium [Cr(VI)], the most toxic chromium species.

Therefore, the chromium-related THQ values should be interpreted cautiously because the analytical measurements represent total chromium rather than chromium speciation. This introduces uncertainty arising from the mismatch between the analytical measurement and the toxicological endpoint. Consequently, the calculated chromium-associated THQ values should be considered preliminary screening-level indicators rather than definitive toxicological risk estimates. Future studies incorporating chromium speciation analysis are necessary for a more accurate assessment of chromium-related health risks.

Overall, the results indicate that honey consumption contributes only a limited proportion to the overall dietary exposure of hazardous trace elements, and the calculated THQ values suggest no significant non-carcinogenic health risk for adult consumers under the evaluated consumption scenarios.

#### Hazard Index

The Hazard Index (HI) values were calculated to evaluate the cumulative non-carcinogenic health risks associated with simultaneous exposure to multiple hazardous trace elements through honey consumption. HI represents the sum of individual THQ values for all investigated elements and is widely used in dietary exposure and food safety risk assessments according to the United States Environmental Protection Agency framework. According to risk assessment guidelines, HI values lower than 1 indicate that the combined exposure is unlikely to cause adverse non-carcinogenic health effects, whereas HI values greater than 1 may indicate potential health concerns.

In the present study, the calculated HI values varied depending on the sampling location and honey consumption scenario (See Table 5). However, after normalization of weekly consumption scenarios to daily exposure equivalents consistent with USEPA methodology, all calculated HI values remained below the threshold value of 1 under both evaluated consumption scenarios. These findings indicate that cumulative exposure to hazardous trace elements through honey consumption is unlikely to pose significant non-carcinogenic health risks for adult consumers.

Among the investigated elements, chromium (Cr) contributed more substantially to the overall HI values compared with the other trace elements. Nevertheless, it is important to emphasize that the chromium-related contribution was estimated using total chromium concentrations obtained by ICP-MS analysis, whereas toxicological reference doses are primarily associated with hexavalent chromium [Cr(VI)], the most toxic chromium species.

Therefore, the chromium-associated HI contributions should be interpreted cautiously because the analytical measurements represent total chromium rather than chromium speciation. This introduces uncertainty arising from the mismatch between the analytical measurement and toxicological endpoint. Consequently, the chromium-related HI values should be regarded as preliminary screening-level indicators rather than definitive toxicological risk estimates. Future studies involving chromium speciation analysis are necessary for a more accurate and toxicologically relevant assessment of chromium-associated health risks.

In contrast, the contributions of other potentially toxic elements such as As, Cd, and Pb to the overall HI values were relatively low, suggesting limited exposure through honey consumption. Similar findings have been reported in previous studies evaluating trace-element contamination in honey and other bee products, where cumulative non-carcinogenic risks generally remained within acceptable limits for consumers [5, 26].

Overall, the present findings demonstrate that honey consumption contributes only a minor proportion to the overall dietary exposure of hazardous trace elements, and the calculated HI values indicate no significant cumulative non-carcinogenic health risk for adult consumers under the evaluated consumption scenarios.

#### Lifetime Cancer Risk

The carcinogenic risk (CR) values associated with hazardous trace-element exposure through honey consumption were evaluated using cancer slope factors (CsF) recommended by the United States Environmental Protection Agency. The calculated CR values were compared with the acceptable risk range of 10⁻⁶–10⁻⁴, which is commonly used in human health risk assessment studies to evaluate potential lifetime carcinogenic risks associated with dietary exposure.

The results indicated that the calculated CR values for most investigated elements and sampling locations remained within the acceptable risk range, suggesting that honey consumption from the investigated districts does not pose a significant carcinogenic health risk for adult consumers (See Table 6). Among the analyzed elements, arsenic (As) exhibited relatively higher CR values compared with the other investigated elements, particularly in honey samples collected from Gürgentepe under the higher consumption scenario. This finding may be associated with the relatively elevated As concentrations detected in this region. Nevertheless, even under the higher consumption scenario, most calculated CR values remained within the acceptable lifetime carcinogenic risk range recommended by USEPA.

For other investigated elements such as Cr, Ni, Pb, and Cd, the calculated CR values were generally low and remained within acceptable safety limits. These findings indicate that the contribution of honey consumption to the overall dietary carcinogenic exposure associated with these elements is limited. Similar observations have been reported in previous studies investigating trace-element contamination in honey and other bee products, which generally concluded that the carcinogenic risks associated with honey consumption remain low and within acceptable safety thresholds [14, 26].

Among the evaluated elements, chromium-related CR values represented one of the major contributors to the overall carcinogenic risk estimates. However, it should be emphasized that the chromium-associated calculations were based on total chromium concentrations determined by ICP-MS analysis, whereas the carcinogenic slope factors used in toxicological risk assessment frameworks are primarily associated with hexavalent chromium [Cr(VI)].

Therefore, the chromium-related CR values should be interpreted cautiously because the analytical measurements represent total chromium rather than chromium speciation. This introduces uncertainty arising from the mismatch between the analytical measurement and toxicological endpoint. Consequently, the calculated chromium-associated CR values should be considered preliminary screening-level indicators rather than definitive carcinogenic risk estimates. Future studies incorporating chromium speciation analysis are necessary for a more accurate and toxicologically relevant evaluation of chromium-associated carcinogenic risk.

Overall, although slight regional variations in carcinogenic risk were observed among the investigated districts, the calculated CR values suggest that honey consumption from the studied regions of Ordu province is unlikely to pose significant carcinogenic health risks for adult consumers. These findings further support the general safety of the analyzed honey samples with respect to hazardous trace-element contamination.

### Multivariate Analysis of Hazardous Trace Elements

**Fig. 3:**
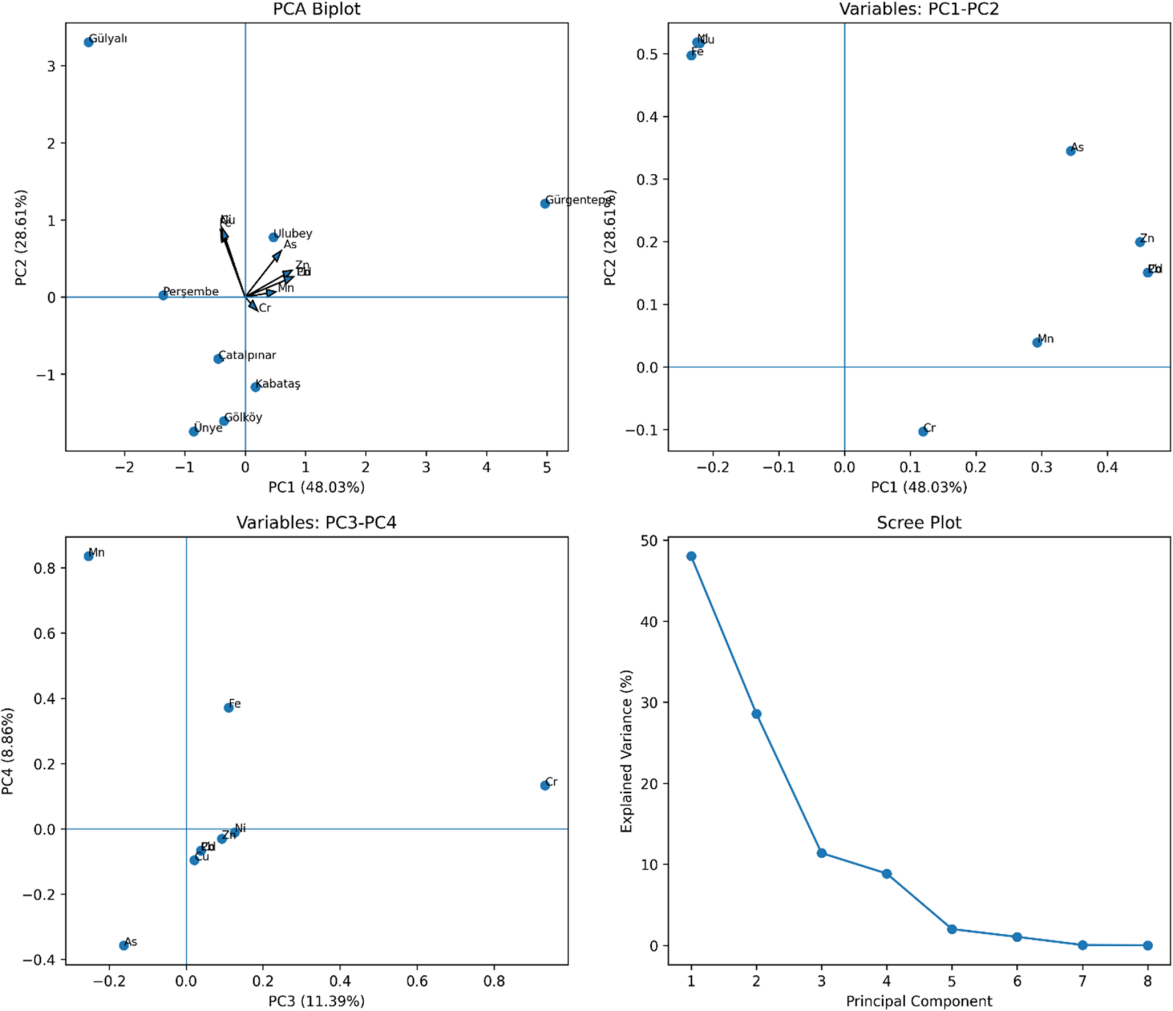
Combined multivariate analysis panel including PCA biplot, variable maps, and scree plot in honey samples

Principal component analysis (PCA) was applied to investigate the relationships among hazardous trace elements in honey samples and to identify their potential sources and presented Fig. 3. PCA is widely used in environmental and food studies as an effective multivariate statistical tool for reducing data dimensionality and revealing hidden patterns in complex datasets [27].

The first two principal components (PC1 and PC2) explained a substantial proportion of the total variance, indicating that the dataset can be effectively interpreted using these components. The PCA biplot (PC1 vs. PC2) revealed clear grouping patterns among the investigated elements. Elements such as Fe, Ni, and Cu exhibited high loadings on PC1, suggesting that these elements are mainly associated with geogenic sources, including soil composition and local geological background. Similar associations have been reported in environmental studies, where Fe and transition metals are linked to natural mineral sources [28].

In contrast, Cd, Pb, and Zn showed strong contributions to PC2 and were closely positioned in the biplot, indicating a common origin. This clustering suggests that these elements are influenced by anthropogenic activities, such as agricultural inputs, atmospheric deposition, and environmental pollution. Previous studies have demonstrated that Cd and Pb are reliable indicators of anthropogenic contamination in environmental and food samples [26]. The PCA score plot further revealed spatial differences among sampling locations, indicating that some districts share similar elemental compositions, whereas others are affected by localized environmental conditions. The PC1–PC2 variable map confirmed the clustering of Fe–Ni–Cu and Cd–Pb–Zn groups, supporting the hypothesis of mixed geogenic and anthropogenic sources.

Although the PC3–PC4 components explained a smaller portion of the total variance, they provided additional insights into secondary interactions among elements, which may be associated with localized environmental factors or minor contamination sources.

Overall, the PCA results demonstrate that the distribution of hazardous trace elements in honey samples is governed by multiple environmental factors, including both natural geological background and human-related activities. These findings are consistent with previous studies and highlight the effectiveness of PCA in identifying the origin and distribution patterns of trace elements in food products [27].

**Fig. 4:**
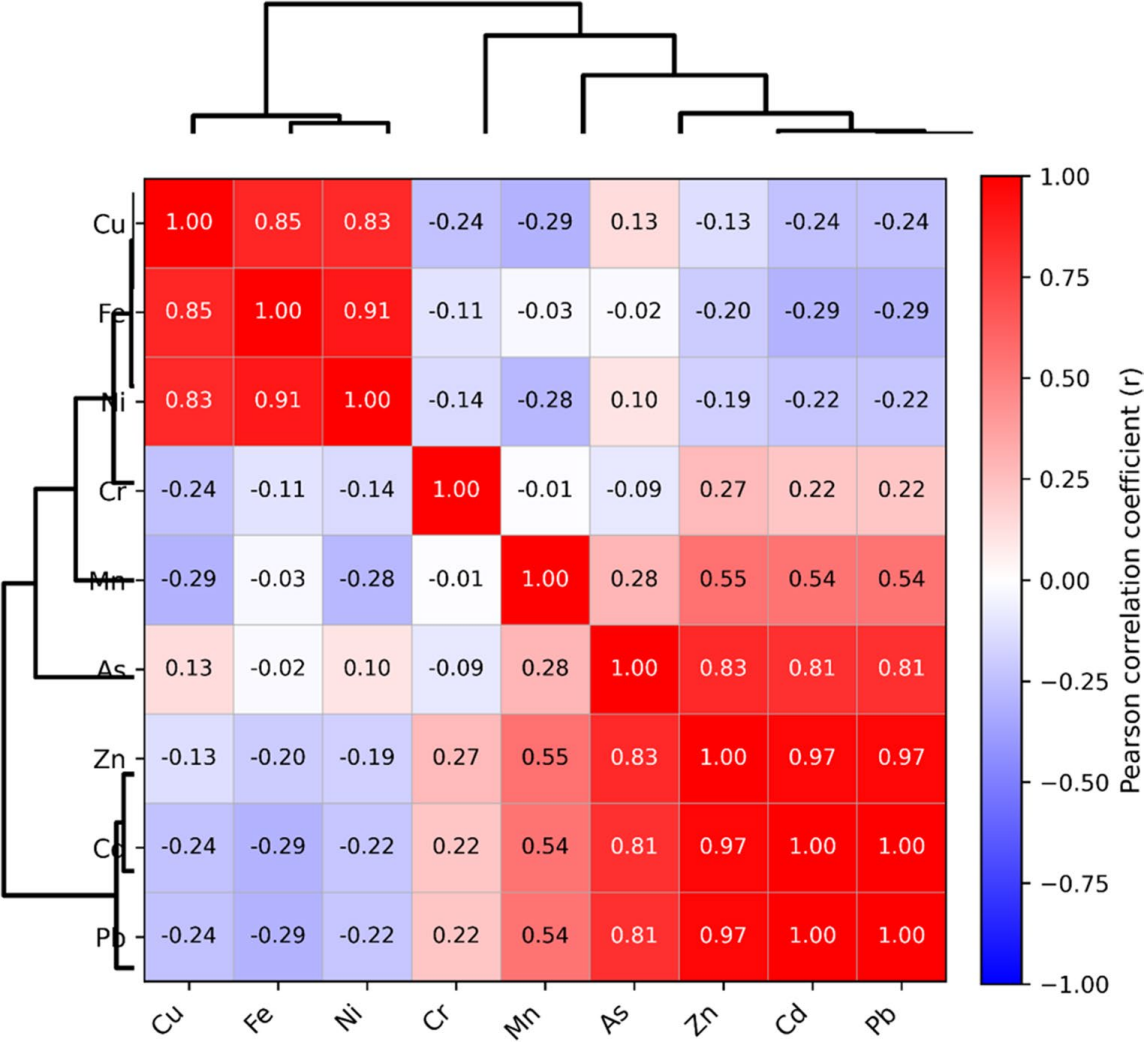
Clustered heatmap of Pearson correlation coefficients among hazardous trace elements in honey samples collected from different districts of Ordu province. The heatmap is combined with hierarchical clustering dendrograms based on correlation distance, illustrating the similarity patterns and potential grouping of elements according to their distribution characteristics

Pearson correlation analysis combined with hierarchical clustering was performed to evaluate the relationships among hazardous trace elements in honey samples and to identify potential similarities in their distribution patterns (See Fig. 4). The results were visualized using a clustered heatmap integrated with dendrograms, which is widely applied in environmental and food chemistry studies to reveal element associations and potential sources [29].

The clustered heatmap revealed distinct grouping patterns among the investigated elements. A strong positive correlation was observed between Cd and Pb, indicating that these elements likely share similar environmental pathways and accumulation mechanisms. This finding suggests a common origin, which is often associated with anthropogenic activities such as agricultural inputs, industrial emissions, and atmospheric deposition [26].

In addition, Zn exhibited strong positive correlations with Cd and Pb, forming a well-defined cluster in both the heatmap and dendrogram. This grouping further supports the hypothesis that these elements are influenced by human-related activities, including the use of fertilizers, pesticides, and environmental pollution. Similar clustering patterns of Zn, Cd, and Pb have been reported in previous studies investigating trace element contamination in food products [5].

Another distinct cluster was observed for Fe, Ni, and Cu, which were closely grouped in the dendrogram. This association suggests a geogenic origin, likely related to soil composition and natural geological background. Transition metals such as Fe and Cu are commonly linked to lithogenic sources, and their co-occurrence is frequently reported in environmental matrices [22].

Elements such as As showed weaker or more isolated relationships, indicating that their distribution may be controlled by localized environmental factors or specific contamination sources. The hierarchical clustering dendrogram clearly separated the elements into distinct groups, reflecting differences in their environmental behavior and origin.

Overall, the combined use of Pearson correlation and hierarchical clustering demonstrates that the distribution of hazardous trace elements in honey samples is governed by multiple environmental processes, including both natural (geogenic) and anthropogenic sources. These results highlight the effectiveness of clustered heatmap analysis as a powerful tool for interpreting complex environmental datasets and identifying potential contamination pathways.

## Discussion

The present study revealed pronounced regional variability in both physicochemical properties and trace element composition of honey samples collected from Ordu Province, highlighting the combined influence of botanical origin, environmental conditions, and local anthropogenic activities. The strong inverse relationship between moisture content and Brix values (*r* = − 0.999) confirms the maturity-dependent concentration effect in honey, whereby reduced moisture levels are associated with increased sugar concentration and enhanced product stability. This relationship is consistent with established honey quality parameters and further supports the reliability of refractometric measurements as a robust indicator of honey maturity and compositional quality [30].

Electrical conductivity exhibited strong positive associations with ash content, pH, and color, indicating that mineral composition is a key determinant of honey characteristics. In particular, darker honey samples with elevated conductivity values, especially those from Gürgentepe and Kabataş, likely reflect increased mineral accumulation associated with soil composition and plant uptake processes. These findings are in agreement with previous studies demonstrating that mineral-rich botanical sources contribute to higher conductivity values and darker honey coloration [31].

Trace element analysis demonstrated clear spatial variability, with certain districts exhibiting relatively elevated concentrations of Fe, Ni, Pb, and Zn. Multivariate analysis revealed that the clustering of Fe–Ni–Cu is indicative of a predominantly geogenic origin, likely linked to local geological background and soil mineral composition. In contrast, the grouping of Cd–Pb–Zn suggests potential anthropogenic inputs, including agricultural practices, traffic emissions, and atmospheric deposition. These findings highlight the dual influence of natural and human-induced factors on the elemental composition of honey and reinforce its applicability as a sensitive bioindicator of environmental quality [15, 23].

Because the analytical measurements were based on total chromium concentrations rather than chromium speciation, whereas the toxicological reference values are primarily associated with hexavalent chromium [Cr(VI)], the chromium-related THQ, HI, and CR estimates should be interpreted cautiously. The calculated values do not represent definitive toxicological risks but rather preliminary screening-level indicators affected by uncertainty arising from the mismatch between analytical measurement and toxicological endpoint. Therefore, chromium speciation analysis is necessary for a more accurate and toxicologically relevant health risk assessment [17, 32].

Despite localized variations in trace element concentrations, the calculated EDI, THQ, HI, and CR values consistently indicate that honey consumption does not pose significant non-carcinogenic or carcinogenic risks under the evaluated scenarios. Even in cases where HI values approached or slightly exceeded the threshold under higher consumption scenarios, the overall contribution of highly toxic elements such as Pb, Cd, and As remained limited, suggesting that honey represents a minor pathway for dietary exposure to these contaminants. Similar findings have been reported in previous risk assessment studies evaluating trace element exposure through food consumption [15].

Overall, the integration of physicochemical characterization, trace element profiling, and chemometric analysis provides a robust and comprehensive framework for differentiating honey samples based on geographical origin and environmental conditions. The findings demonstrate that honey not only represents a high-value natural food product but also serves as an effective indicator of environmental quality, reflecting both geogenic and anthropogenic influences. This integrated approach further strengthens the use of honey as a reliable tool in environmental monitoring and food safety assessment [22].

To enhance the contextual significance of the findings, the obtained trace element concentrations were compared with recent studies conducted in different geographical regions. Compared to honeys from industrialized regions in Europe and Asia, the concentrations of Pb and Cd observed in the present study were generally lower, indicating relatively limited anthropogenic pressure in the studied areas. For instance, studies conducted in Italy and China reported higher Pb and Cd levels, attributed to industrial emissions and urbanization.

In contrast, the relatively elevated Fe and Ni concentrations observed in certain districts of Ordu Province appear to be consistent with geogenic enrichment patterns reported in regions with mineral-rich soils. Similar geogenic signatures have been reported in honey samples from mountainous regions, where soil composition strongly influences elemental transfer to plants and subsequently to honey.

Furthermore, temporal comparisons with earlier studies conducted in Türkiye indicate that trace element levels in honey have remained relatively stable over time, suggesting that large-scale environmental contamination has not significantly increased in the studied region. This temporal consistency reinforces the reliability of honey as a bioindicator for long-term environmental monitoring.

Overall, these comparative insights highlight that the trace element profile of honey from Ordu Province reflects a balanced influence of natural geological background and limited anthropogenic impact, distinguishing it from more heavily industrialized regions.

The comparative evaluation presented in Table 7 clearly demonstrates that the concentrations of toxic elements such as As, Cd, and Pb in honey samples from Ordu Province are generally lower than those reported in heavily contaminated regions, such as Romania and Ethiopia, where significant anthropogenic pressure has been documented. In contrast, the observed levels are more comparable to those reported in relatively less polluted regions such as Italy and the United States [22, 33–42].

**Table 7:** Comparison of hazardous trace element concentrations (mg/kg) in honey from Ordu Province with recent literature data from different geographical regions

Study	Location	Method	As (mg/kg)	Cd (mg/kg)	Pb (mg/kg)	Cr (mg/kg)	Ni (mg/kg)	Zn (mg/kg)	Fe (mg/kg)	Key Findings/Comparison
This study	Ordu, Türkiye	ICP-MS	Low	Low	Low–moderate (Gürgentepe ↑)	Relatively influential in risk	Moderate–high (Gülyalı ↑)	Moderate (Ulubey ↑)	High (Gülyalı ↑)	Low toxic metals; Cr dominates risk indices; mixed geogenic–anthropogenic sources
Naccari et al., 2025 [22]	Italy	ICP-MS	0.0026–0.0128	0.0063–0.0090	0.079–0.125	–	–	–	–	Comparable low As–Cd; Pb slightly higher than this study
Vlad et al., 2025 [33]	Romania	FAAS/GFAAS	–	0.02–0.37	0.72–1.69	–	–	0.91–1.93	–	Much higher Pb and Cd → strong anthropogenic pollution
Godebo et al., 2025 [34]	USA	ICP-MS	0.0038	0.00075	0.0080	0.0196	0.0295	–	–	Lower global baseline; supports low contamination level
Kastrati et al., 2023 [35]	Kosovo	Multi-method	0.0068	0.023	0.20	0.38	0.32	2.8	4.6	Higher Cr and Pb; similar geogenic patterns (Fe–Ni)
Çakmak & Tonbak, 2026 [36]	Türkiye	ICP-OES	–	–	0.037–0.067	0.513–0.661	–	–	–	National comparison; Cr values similar magnitude
Teferi et al., 2026 [37]	Ethiopia	ICP-OES	–	1.38–2.30	–	–	6.05–8.85	75.7–209.5	–	Extremely high metals → strong environmental contamination
Inaudi et al., 2025 [38]	Italy	ICP-OES/HR-ICP-MS	< LOQ	< 0.00323	< LOQ – 0.300	0.017–0.139	0.0036–0.272	1.517	5.58	Generally low toxic metal levels; Pb variability indicates localized contamination patterns
Squadrone et al., 2020 [39]	Italy	ICP-MS	0.0077–0.017	< 0.001	0.012–0.071	0.067–0.093	0.10–0.22	0.91–1.5	1.1–2.0	Comparable Pb and Cr levels; lower Cd concentrations than the present study
Bora et al., 2024 [40]	Romania	ICP-MS	< LOQ	0.007	0.038	0.201	0.034	2.507	4.316	Moderate trace metal levels; Cr and Zn slightly higher than in Ordu samples
Kılıç Altun et al., 2017 [41]	Turkey	ICP-MS	–	< 0.001	< 0.001	< 0.001	< 0.001	0.049	0.268	Very low contamination profile; substantially lower trace element concentrations overall
Karlıdağ & Kolaylı, 2025 [42]	Türkiye (Malatya/Yanmadağ)	ICP-MS	0.12	0.05	0.10	0.46	0.03	0.62	0.41	Higher As and Cr concentrations; possible influence of regional geological background
Karlıdağ & Kolaylı, 2025 [42]	Türkiye (Malatya/Battalgazi)	ICP-MS	–	–	0.11	0.21	0.14	1.30	7.55	Elevated Fe and Pb levels suggest mixed geogenic and anthropogenic influences

A notable distinction of the present study is the relatively higher contribution of chromium to non-carcinogenic risk indices (THQ and HI), despite its moderate concentration levels. This observation is consistent with previous studies reporting elevated Cr values; however, it should be emphasized that risk calculations based on total chromium may overestimate actual toxicity due to the predominance of Cr(III) in food matrices.

Furthermore, the enrichment of Fe and Ni in specific districts supports a geogenic origin, while localized increases in Pb and Zn suggest anthropogenic influences, consistent with patterns reported in Kosovo and other regional studies. These findings confirm that honey from Ordu reflects a relatively low contamination burden overall, while still capturing localized environmental signatures, highlighting its effectiveness as a bioindicator of environmental quality.

## Conclusion

This study provided a comprehensive evaluation of the physicochemical properties, trace element composition, and associated health risks of honey samples collected from different districts of Ordu province, Türkiye. The results revealed clear regional variations in both physicochemical parameters and trace element concentrations, highlighting the significant influence of geographical origin, environmental conditions, and botanical diversity on honey composition. Strong relationships among key parameters, particularly between moisture content, Brix, electrical conductivity, and color, were confirmed through correlation and multivariate analyses, emphasizing the role of mineral content and compositional factors in determining honey quality. Despite the observed variability, all physicochemical parameters remained within internationally accepted limits, indicating that the analyzed honey samples meet quality standards. Trace element analysis demonstrated spatial differences, with certain regions showing relatively elevated levels of specific elements; however, risk assessment indices, including EDI, THQ, HI, and CR, indicated that honey consumption does not pose significant non-carcinogenic or carcinogenic health risks under the evaluated consumption scenarios. Overall, the findings confirm that honey from Ordu province is safe for consumption and exhibits distinct regional characteristics. The integration of physicochemical analysis, trace element determination, and chemometric approaches proved to be an effective strategy for honey characterization and environmental assessment. These results contribute to the understanding of honey quality and support the use of honey as a potential bioindicator of environmental conditions. Continuous monitoring of trace elements in honey is recommended to ensure food safety and to track environmental changes in apicultural regions.

## Data Availability

The datasets generated during and/or analyzed during the current study are available from the corresponding author on reasonable request.
